# Growth performance, organs weight, intestinal histomorphology, and oocyst shedding in broiler chickens offered novel single strain *Bacillus subtilis* isolated from camel dung and challenged with *Eimeria*

**DOI:** 10.1016/j.psj.2024.103519

**Published:** 2024-02-07

**Authors:** Alison Gelinas, Sudhanshu Sudan, Rob Patterson, Julang Li, David Huyben, John R. Barta, Elijah G. Kiarie

**Affiliations:** ⁎ Department of Animal Biosciences, University of Guelph, Guelph, ON, N1G 2W1, USA; † CBS BioPlatforms Inc., Calgary, AL, T2C 0J7, USA; ‡ Department of Pathobiology, University of Guelph, Guelph, ON, N1G 2W1, USA

**Keywords:** broiler chicken, *Eimeria* and coccidiosis, growth performance and intestinal health, single strain *Bacillus subtilis* (BS-9) direct-fed microbial

## Abstract

We evaluated a single strain *Bacillus subtilis* BS-9 direct-fed microbial (**BSDFM**) isolated from camel dung in *Eimeria* challenged broiler chickens. Seven-hundred d-old Ross 708 male chicks were placed in pens (25 birds/pen) and allocated to 2 treatments (n = 14). From d 0 to 13, control pens received untreated water (-BSDFM), and 2 treated pens received water and 2 mL x 10^8^ colony forming unit/bird/d (+BSDFM); daily water intake (**WI**) was recorded. On d 9, birds in half (+*Eimeria*) of pens per treatment received of 1 mL of *Eimeria maxima* and *Eimeria acervulina* oocysts orally, and the other half (*-Eimeria*) sterile saline solution. Birds had *ad libitum* access to feed and a water line from d 14. Feed intake (**FI**), body weight (**BW**) and mortality were recorded for calculating BW gain (**BWG**) and feed conversion ratio (**FCR**). On d 14 and 35, samples of birds were necropsied for organ weight and intestinal measurements. Excreta samples were collected from d 14 to 19 for oocyst count. There was no treatment effect (*P* > 0.05) on growth performance or WI on d 0 to 9. There were interactions between BSDFM and *Eimeria* on d 19 (*P* = 0.014) and 29 (*P* = 0.036) BW with unchallenged +BSDFM birds being heavier than birds in the other treatments. The main effects (*P* < 0.05) on d 10 to 35 FI, BW, and BWG were such that +BSDFM increased and *Eimeria* decreased (*P* < 0.01) these parameters. There was interaction (*P* = 0.022) between BSDFM and *Eimeria* on d 10 to 35 FCR such that the FCR of challenged -BSDFM birds was poor than that of unchallenged counterparts, but none differed with +BSDFM birds. There was an interaction (*P* = 0.039) between BSDFM and *Eimeria* on d 14 bursa weight with challenged birds exhibiting heavier bursa than unchallenged +BSDFM birds. *Eimeria* reduced (*P* = 0.01) and BSDFM (*P* = 0.002) increased the villi height to crypt depth ratio. Results showed that BSDFM supplementation via water can support the growth performance of broiler chickens challenged with *Eimeria* and may be a strategy to reduce adverse effects of coccidiosis.

## INTRODUCTION

Poultry accounts for over 30% of global meat production and is expected to increase by 55% (Alexandratos and Bruinsma, 2012) to meet the growing demand by 2050 (Mottet and Tempio, 2017). Optimal broiler chicken growth requires a functional gastrointestinal tract (**GIT**) to digest and absorb nutrients, domicile stable microbiota, and provide host protection (Aliakbarpour et al., 2012; Kiarie et al., 2013; Kiarie et al., 2019; Kogut, 2019; Mak et al., 2022). Early broiler growth and the establishment of a functional GIT are becoming increasingly important attributes due to the shortening broiler production cycle. Preferential small intestinal (**SI**) development during the first ten d of broiler chicks allows for optimal nutrient absorption later in life (Wijtten et al., 2012).

Coccidiosis, caused by *Eimeria* species protozoan parasites, is an inherent costly risk in poultry production and has been estimated to cost the global poultry industry $14 billion (USD) annually (at 2016 prices) (Blake et al., 2020; Poudel et al., 2021). The poultry industry has option of using drugs or vaccines to control coccidiosis. With respect to vaccines, the principle is to induce host immunity against later homologous *Eimeria* spp. infections (Gautier et al., 2020; Soutter et al., 2020). However, vaccinations with even low doses of *Eimeria* oocysts cause intestinal lesions, altering the intestinal morphology and triggering intestinal inflammation (Ritzi et al., 2014, 2016; Kiarie et al., 2019; Attree et al., 2021). This damage during early development impairs intestinal barrier function and development, resulting in bacterial translocation and increased risk of secondary bacterial infections such as Necrotic enteritis (**NE**) (Honda and Littman, 2012; Poudel et al., 2021). Preventative antibiotics or growth promoters (**AGP**) have been conventionally used to control coccidiosis and later NE while protecting the growth performance of birds (Bean-Hodgins and Kiarie, 2021; Mak et al., 2022). However, there is growing restrictions on AGP use and the global shift towards antibiotic-free animal production threaten the global food supply chain. Therefore, new strategies in place of AGP must be implemented to maintain the growth performance of broiler chickens.

The consensus defines probiotics as “live microorganisms that when administered in adequate amounts confers health benefit on the host” (Hill et al., 2014). Albeit variability in some cases, *B. subtilis* based probiotics or direct fed microbials (**BSDFM**) have been shown to enhance growth performance, nutrient digestibility, indices of intestinal health and decrease in the abundance of *Clostridium perfringens* and other pathogenic bacteria in the gut (Waititu et al., 2014; Rhayat et al., 2017; Wang et al., 2018; Mohammadigheisar et al., 2019; Neijat et al., 2019a,b,c; Wang et al., 2021; Akhtar et al., 2022). These attributes make BSDFM a promising alternative to AGP for controlling coccidiosis and secondary bacterial infections in poultry. Moreover, the spore-forming capability of *B. subtilis* confers benefits against chemical and physical stressors associated with feed manufacturing and the gastrointestinal tract (**GIT**) (Rhayat et al., 2017; Grant et al., 2018; Latorre et al., 2018; Zhao et al., 2020; Sudan et al., 2021). In addition to modulation of the GIT microbiota via competitive exclusion, *B. subtilis* contributes to animal health through the production of fatty acids, enzymes, metabolites, vitamins, and antimicrobial peptides (**AMP**) that are cytotoxic to pathogenic bacteria (Cai et al., 2015; Elshaghabee et al., 2017; Grant et al., 2018; Attree et al., 2021).

The multitude of different *B. subtilis* strains vary in their production of enzymes and AMP, and thus, the potential to modulate the gut microbiota and, later, animal performance. We recently characterized a high cellulase and protease producing single strain *B. subtilis* that could survive wide temperature range, pH conditions, and bile conditions, was cytotoxic to pathogenic bacteria, conferred cytoprotection to swine epithelial cells under an enteric microbial pathogen challenge and produced unique AMP during *in vitro* co-cultures (Sudan et al., 2021, 2022; Akhtar et al., 2022). When provided via the waterline twice daily to piglets for 21 d at 2 mL × 10^7^ or × 10^9^ CFU, the *B. subtilis* strain improved growth performance in piglet and various markers of intestinal health (Sudan et al., 2023). Based on the prior *in vivo* and *in vitro* work, we hypothesized that the novel *B. subtilis* strain would improve broiler chicken intestinal health and growth performance parameters. Additionally, the boost in intestinal development from strain would decrease the adverse effects of coccidiosis. Therefore, the objective of the current study was to evaluate the effects of single strain *B. subtilis* (BSDFM) when provided via the water on growth performance, water intake, organ weight, intestinal histomorphology, and oocyst shedding in broiler chickens challenged with *Eimeria*.

## METHODS AND MATERIALS

The animal utilization protocol (#4403) for this experiment was approved by the University of Guelph Animal Care Committee, and all birds were cared for per the Canadian Council on Animal Care Guidelines (CCAC, 2009).

### Bacillus Subtilis Preparation and Dosing

The single strain *Bacillus subtilis* (BSDFM) originally named CP-9, now renamed BS-9, was isolated from camel feces, and characterized at the University of Guelph (Akhtar et al., 2022). The BSDFM production and preparation followed procedures were described by Sudan et al. (2023). Frozen BSDFM stock was taken from the -80⁰C freezer and thawed at room temperature. The BSDFM culture was prepared on fresh Luria-Bertani (LB, St. Louis, MO) agar plates and incubated at 37⁰C overnight with constant shaking. A single colony was selected and inoculated in 100 mL LB broth medium in an incubator shaker (New Brunswick Scientific, Enfield, CT) at 200 RPM, 37⁰C for 18 h under aerobic conditions. To confirm BSDFM enumeration and growth, the optical density was measured. Water samples from the Arkell Poultry Research Station were collected and used to plate BSDFM to examine its viability in the drinking water. The BSDFM stock for the animal trial was prepared to contain ∼1×10^8^ colony-forming units (CFU) of BSDFM/mL. A total of 700 mL fresh batches of broth were prepared daily for application and packed in two 350 mL media bottles. The BSDFM was applied in drinking water from d 0 to 13 of broiler chicken life. The target dose was 2 mL of BSDFM stock per bird per day mixed with the estimated daily water intake (**WI**) per birds. The WI was calculated based on the performance objectives of Ross-708 daily feed intake (**FI**) guidelines for male chicks (Aviagen, 2019) and the published ratio of WI and FI (∼1.77g/g) according to Pesti et al. (1985). Due to the risk of leakage, drip loss, and the requirement for a minimal addition of water to cover the drinker nipples, an additional 10% of the daily estimated WI was used as a buffer to the calculated WI to ensure no out-of-water events.

### Bird Housing, Care, and Diets

A total of 700-day-old male Ross x Ross 708 broiler chicks were obtained from a hatchery (Maple Leaf Foods, New Hamburg, ON, Canada). Upon arrival at the Arkell Poultry Research Station (Guelph, ON), birds were sorted by BW and placed in 28-floor pens (25 birds/pen). The pens were equipped with a hanging feeder and water line with drinking nipples. The water line was lifted beyond the reach of chicks in the first 13 d to allow for the application of BSDFM in hanging nipple drinkers. The room temperature was initially set at 32⁰C on d 0 and gradually reduced to 21⁰C by d 30. The lighting program was 23 h of light (20 + LUX) on d 0, with a gradual decrease in hours of light until d 4, then 16 h of light (10–15 LUX) from d 4 until the end of the trial. A basal corn and soybean meal-based diet was formulated to meet the breeder requirements (Table 1) in a 2-phase feeding program: starter (d 0–13) and grower (d 14–35). Diets were prepared at the Arkell Feed Mill (Guelph, ON, Canada) as a crumble for the starter and pellet for the grower.

**Table 1:** Composition of the diets (%, as fed).

Ingredient, %	Starter (d 0–13)	Grower-finisher (d 14–35)
Corn	46.0	55.0
Soybean meal	35.1	24.4
Wheat	10.0	10.0
Soy oil	2.72	3.02
Pork meal (58%)	2.00	5.00
Monocalcium phosphate	1.64	0.68
Limestone	0.86	0.24
Vitamins and trace minerals premix^1^	0.50	0.50
DL-Methionine	0.37	0.32
L-Lysine HCL	0.32	0.31
Salt	0.24	0.24
L-Threonine	0.19	0.21
Sodium bicarbonate	0.12	0.07
Total	100.0	100.0
Calculated provisions		
AME, kcal/kg	3,000	3,150
Crude protein^2^, %	23.0	20.5
SID Lysine, %	1.28	1.09
SID Met + Cys, %	0.96	0.84
SID Methionine, %	0.67	0.59
SID Threonine, %	0.87	0.74
SID Tryptophan, %	0.26	0.21
Calcium, %	0.96	0.84
Available phosphorus, %	0.48	0.42
Sodium, %	0.16	0.16
Chloride, %	0.23	0.23
Analyzed provisions		
Gross energy, kcal/kg	4,030	4,165
Crude protein, %	21.9	19.2
Crude fat, %	5.23	6.16
Starch, %	33.9	39.4
Calcium, %	0.94	0.81
Total phosphorus, %	0.82	0.67
Sodium, %	0.19	0.18

1 Provided per kg of the diet: vitamin A, 1,200 KIU; vitamin D_3_, 500 KIU; vitamin E, 8,000 IU; vitamin B_12_, 1,700 mcg; biotin, 22 mg; menadione, 330 mg; thiamine, 400 mg; riboflavin, 860 mg; pantothenic acid, 2,000 mg; pyridoxine, 430 mg; niacin, 6,500 mg; folic acid, 220 mg; choline, 60,000 mg; manganese, 7000mg; iron, 6000 mg; copper, 1000 mg; zinc, 7000 mg; iodine, 100 mg; selenium, 30mg

2 Crude protein, N x 6.25%.

### Experimental Procedures

*The BSDFM application:* On d 0, the pens were allocated to 2 water treatments, -BSDFM or +BSDFM, to give 14 replicate pens per treatment. Distribution of pens within the room was such that one side was -BSDFM and the other +BSDFM to minimize cross-contamination. From d 0 to 13, the expected WI per pen was calculated based on pen population to facilitate the calculation of the volume of water to be treated with BSDFM. The total daily estimated WI (including buffer) for +BSDFM pens was split for application such that birds received 40% in the morning and 60% in the afternoon. Corrections and adjustments were made based on the prior day WI if needed (for example, larger than anticipated leftover). A large plastic container was used to mix each portion with 350 mL of BSDFM stock and dispensed in bell drinkers assigned to each pen. The amount of treated water per pen was measured using graduated cylinders based on the pre-calculated WI. Morning and afternoon treatment, similar to that of Sudan et al. (2023), was an attempt to ensure the freshness and viability of BSDFM stock. Control pens were provided with fresh water daily in the morning. The water remaining in the nipple drinker was recorded every morning for 13 d to facilitate the calculation of WI.

*Eimeria Challenge*: On d 9, birds in half of the replicate pens per treatment received oral gavage (+*Eimeria*) of 1 mL of *E. maxima* (25,000 oocysts) and *E. acervulina* (100,000 oocysts), and the other half (-*Eimeria*) received an equal volume of sterile saline solution. This effectively created a 2 × 2 factorial treatment arrangement for the postchallenge period (d 10–35) with BSDFM and *Eimeria* as the main effects (7 replicate pens per treatment). The separation of rows based on treatment and challenge and a biosecurity floor plan where daily health checks followed a marked path was in place to reduce cross-contamination between treatment groups. The *Eimeria* culture and dosage have been validated in previous studies in our laboratory (Kim et al., 2017; Akbari Moghaddam Kakhki et al., 2019; Leung et al., 2019a,b; Lu et al., 2019; Kim et al 2022).

### Sampling Procedures

**Figure 1:**
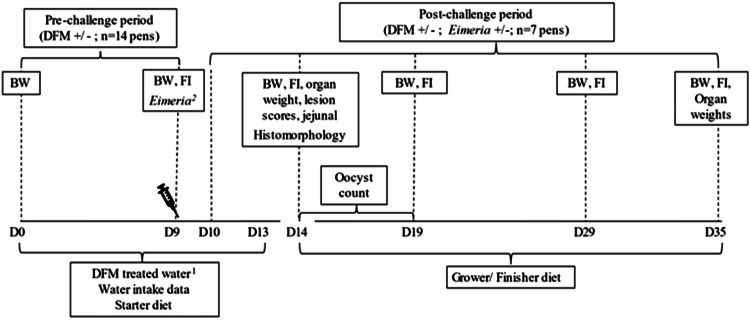
Experimental procedure timeline. ^1^+BSDFM birds received 2 mL of broth containing 10^8^ CFU of BSDFM per mL in drinking water daily from d 0 to 13. ^2^*Eimeria* birds received 1 mL of saline solution with 25,000 *E. maxima* and 100,000 *E. acervulina oocysts (+Eimeria)* or the same solution without *Eimeria (-Eimeria)* on d 9.

Body weight (**BW**) and feed intake (**FI**) were recorded on a pen basis on d 0, 9, 14, 19, 29, and 35 to calculate pre- (d 0–9) and postchallenge (d 10–35) BW gain (**BWG**) and feed conversion ratio (**FCR**) (Figure 1). Mortalities were recorded daily, and birds were culled from the study and recorded as mortalities only if they appeared unhealthy and to relieve suffering. The mortalities and removed birds were used for correcting FCR. On d 14, 4 birds per pen were weighed, euthanized by cervical dislocation, and necropsied. The liver, spleen, and bursa were dissected, blotted dry, and weight recorded. The jejunum was located and removed at the duodenal loop and 2 cm anterior to the Markel diverticulum (Kim et al., 2017). Mid-jejunal samples (∼2 cm) were immediately placed and stored in 10% formalin for histomorphology analyses. Intestinal regions (duodenum and jejunum) were removed and blindly assessed for *Eimeria* lesion scores using a scale of 0 (no lesions) to 4 (high lesions) (Johnson and Reid, 1970). On d 35, 2 birds per pen were euthanized, as described for d 14, and the liver, bursa, spleen, and breast were weighed. Fresh excreta samples were collected from each pen d 14 to 19 (i.e., 5–10 d postchallenge) and placed in 50 mL centrifuge containers filled with pre-weighed potassium dichromate and stored (4 ⁰C) until required for oocysts count.

### Sample Processing and Laboratory Analyses

Starter and grower/finisher diets were ground finely. Samples were analyzed at a commercial laboratory (SGS Canada Inc, Guelph, ON, Canada) for dry matter, crude protein, crude fat, starch, and minerals (calcium, and phosphorus) using Association of Official Agricultural Chemists methods (930.15, 935.11, 920.39, 920.40, 935.12, respectively) (AOAC, 2005). The gross energy of samples was determined by bomb calorimetry (IKA Calorimeter System C 6000; IKA Works, Wilmington, NC) at the University of Guelph, ON, Canada. Jejunal samples were prepared for histology slides within 48 h of collection via slicing circular, longitudinal sections from each and pooling the cut pieces by pen onto cassettes. The cassettes were sent to AHL (Animal Health Laboratory, University of Guelph) for histological slides preparation. Briefly, the jejunal samples were fixed, embedded in paraffin, partitioned (5µm), and dyed with hematoxylin and eosin (Kiarie et al., 2018). Once obtained, slides were read under a Leica DMR microscope (Leica Microsystems, Wetzlar, Germany) at 5x magnification. The villi height (**VH**) and crypt depth (**CD**) (µm) were recorded at 15 different areas (if visible) and averaged per pen. The VH was divided by CD to calculate the VH: CD ratio. The number of oocysts was determined using the McMaster counting chamber technique with saturated NaCl as the floatation medium (Price et al., 2014).

### Calculations and Statistical Analyses

The organs (liver, spleen, bursa, and breast) weight were expressed in g/kg BW. To determine oocyst counts, the slides were counted twice, and the 2 counts were averaged to get a single mean count. The mean count, dilution factor, sample volume, and fresh sample weight were then entered into the following formula to calculate oocysts per gram of excreta:

$$Oocysts\;per\;ml\;in\;sample=\;\frac{\left(Chamber\;count\;1\;oocyst+Chamber\;count\;2\;oocyst\right)\times Dilution\;Factor\;}{0.3\;ml\;chamber\;volume\;counted}$$

$$Oocysts\;per\;gram\;\left(OPG\right)=\;\frac{oocyst\;per\;ml\;\left(sample\right)\times sample\;volume\;}{fecal\;sample\;dry\;weight\;\left(g\right)}$$

Outliers in growth performance data were first identified using box plot procedures of excel and confirmed using the PROC SGPLOT procedure of SAS Studio version 9.4. Outliers were removed if beyond 10% of ± 1.5 (IQR) of Q_1_ or Q_3_, respectively. The pen was the experimental unit in the statistical analyses. The data were subjected to the PROC GLIMMIX procedures of SAS version 9.4 for LSmeans, using Tukey method. The BSDFM was the fixed factor for the pre-challenge (d 0–9) period. In the post-challenge (d 10–35) period, BSDFM, *Eimeria*, and their interactions were the fixed factors. Residuals of the data were analyzed for normality, with water intake, growth performance, and organ weight data showing a normal distribution. Oocyst shedding data showed a Poisson distribution, and data were log_10_ transformed and then analyzed as repeated measures with BSDFM, time (i.e., day), *Eimeria*, and their interactions as fixed factors. No lesions were recorded in non-challenged birds on d 14 except for 2 birds; thus, lesion scores were only analyzed in challenged birds, with BSDFM as a fixed factor. Differences were considered statistically significant when *P* ≤ 0.05 or a tendency when *P* < 0.10.

## RESULTS

### Growth Performance and Water Intake

In the pre-challenge period (d 0–9), BSDFM had no (*P* > 0.05) effect on growth performance, WI, and the WI: FI ratio (Table 2). Growth performance and WI for the postchallenge period are shown in Table 3. There were no effects of BSDFM, *Eimeria*, or their interactions on mortality in the postchallenge (d 10–35) period (data not shown). There was no (*P* > 0.05) interaction between BSDFM and *Eimeria* on WI and WI: FI ratio between d 10-14 of life (d 1–5 postchallenge). However, *Eimeria* increased (*P* = 0.029) WI: FI ratio by 11.1%. Interactive effects between BSDFM and *Eimeria* were observed for growth performance parameters at time points between d 10 and 35 (Table 3). There was interaction (*P* = 0.048) between BSDFM and *Eimeria* on d 10-14 FI where unchallenged BSDFM birds ate more than birds in the other treatments. There were interactions between BSDFM and *Eimeria* on d 19 (*P* = 0.014) and 29 (*P* = 0.036) with unchallenged BSDFM birds being heavier than birds in other treatments. The main effects (*P* < 0.05) on d 10-35 FI, BW and BWG were such that BSDFM increased and *Eimeria* decreased (*P* < 0.01) these parameters. The interaction (*P* = 0.021) between BSDFM and *Eimeria* on d 10 to 14 FCR was such that challenged control birds had poorer FCR compared to BSDFM birds. There was interaction (*P* = 0.022) between BSDFM and *Eimeria* of d 10 to 35 FCR such that challenged control birds had poor FCR relative to unchallenged control birds, but none differed with BSDFM birds.

**Table 2:** Effect of single strain *B. subtilis* direct fed microbial (BSDFM) supplementation in drinking water on growth performance and water intake in broiler chickens fed a corn and soybean meal-based diet (prechallenge period).

Item	-BSDFM	+BSDFM^1^	SEM	*P*-value
Initial body weight (BW), g/ bird	40.7	40.7	0.06	0.611
BW, g/bird, d 9	194.9	198.9	3.18	0.381
BW gain (BWG), g/bird, d 0-9	154.1	158.2	3.19	0.377
Feed intake (FI), g/bird, d 0-9	187.4	187.2	2.67	0.958
Feed conversion ratio (FCR), g/g, d 0-9	1.26	1.27	0.031	0.860
Water intake (WI), mL/ bird, d 0-9	441.5	430.2	8.07	0.330
WI: FI ratio, d 0-9	2.33	2.28	0.042	0.422

1 +BSDFM birds received 2 mL of broth containing 10^8^ CFU of BSDFM per mL in drinking water daily from d 0 to 13.

**Table 3:** Effects of single strain *B. subtilis* direct fed microbial (BSDFM) supplementation in drinking water on water intake and growth performance in broiler chickens fed a corn and soybean meal-based diet challenged with *Eimeria* (postchallenge period).

	Interaction effects					Main effects					*P*-values		
BSDFM^1^	-		+			BSDFM		*Eimeria*					
*Eimeria*^2^	-	*+*	-	*+*	SEM	-	+	-	*+*	SEM	BSDFM	*Eimeria*	BSDFM*Eimeria
Water intake, mL/ bird													
d 10–13	335	347	357	339	8.26	341	348	346	343	7.00	0.510	0.766	0.144
Water intake: Feed intake ratio													
d 10–13	1.50	1.61	1.40	1.62	0.071	1.56	1.51	1.45^b^	1.62^a^	0.05	0.507	0.029	0.458
Feed intake, g/ bird													
d 10–14	224^b^	216^b^	256^a^	213^b^	8.35	220	234	240^a^	214^b^	5.9	0.095	0.006	0.048
d 15–19	396	290	384	276	14.04	343	330	390^a^	283^b^	9.9	0.363	<0.001	0.918
d 20–29	,1133	1,098	1,190	1137	25.09	1,116	1,163	1,161	1,118	17.7	0.071	0.094	0.725
d 30–35	926	919	962	933	12.63	923	947	944	926	8.9	0.062	0.160	0.377
d 10–35	2,650^b^	2,523^c^	2,791^a^	2,552^bc^	32.33	2,586^b^	2,671^a^	2,721^a^	2,537^b^	22.9	0.016	<0.001	0.096
Body weight, g/ bird													
d 14	437	388	456	395	7.5	412	426	447^a^	392^b^	5.3	0.088	<0.001	0.429
d 19	644^b^	542^c^	709^a^	551^c^	10.7	593^b^	630^a^	677^a^	546^b^	7.6	0.002	<0.001	0.014
d 29	1,441^b^	1,334^c^	1,545^a^	1,369^c^	15.4	1387^b^	1,457^a^	1,493^a^	1,351^b^	10.9	<0.001	<0.001	0.036
d 35	1,866	1,758	1,959	1,790	21.7	1812^b^	1,875^a^	1,913^a^	1,774^b^	15.4	0.008	<0.001	0.176
Body weight gain, g/ bird													
d 10–14	251	184	258	196	4.4	218^b^	227^a^	254^a^	190^b^	3.1	0.042	<0.001	0.632
d 15–19	198^a^	142^b^	232^a^	139^b^	9.1	170	186	215^a^	141^b^	6.5	0.100	<0.001	0.052
d 20–29	797	792	836	819	10.8	795^b^	827^a^	817	805	7.6	0.006	0.305	0.589
d 30–35	425	418	414	421	11.0	421	418	419	419	7.8	0.764	0.997	0.529
d 10–35	1678^a^	1552^b^	1761^a^	1591^b^	21.3	1616^b^	1676^a^	1720^a^	1572^b^	15.1	0.010	<0.001	0.331
Feed conversion ratio, g/g													
d 10–14	0.892^c^	1.168^a^	0.992^bc^	1.109^ab^	0.032	1.030	1.050	0.942^b^	1.138^a^	0.023	0.531	<0.001	0.021
d 15–19	1.803	2.083	1.658	2.063	0.087	1.943	1.861	1.731^b^	2.073^a^	0.061	0.352	<0.001	0.477
d 20–29	1.448	1.413	1.437	1.4013	0.017	1.430	1.419	1.443^a^	1.407^b^	0.012	0.507	0.044	0.985
d 30–35	2.325	2.208	2.326	2.216	0.059	2.267	2.271	2.325	2.212	0.042	0.945	0.069	0.960
d 10–35	1.574^b^	1.630^a^	1.586^ab^	1.580^ab^	0.012	1.601	1.583	1.580	1.604	0.009	0.153	0.060	0.022

a,b,c Within a row, LSMeans assigned different letters differ, *P* < 0.05.

1 +BSDFM birds received 2 mL of broth containing 10^8^ CFU of BSDFM per mL in drinking water daily from d 0-13.

2 *Eimeria* birds received 1 mL of saline solution with 25,000 *E. maxima* and 100,000 *E. acervulina oocysts (+Eimeria)* or the same solution without *Eimeria (-Eimeria)* on d 9.

### Organ Weight, Intestinal Histomorphology, Lesion Scores and Oocyst Shedding

There were no (*P* > 0.05) interactions between BSDFM and *Eimeria* or main effects of BSDFM on liver and spleen weight in 14- and 35-day-old broiler chickens (Table 4). *Eimeria* increased d 14 liver (*P* < 0.001) and d 35 spleen (*P* = 0.011) weights, respectively. There was an interaction (*P* = 0.039) between BSDFM and *Eimeria* on d 14 bursa weight such that *Eimeria* birds exhibited heavier bursa than unchallenged BSDFM birds. However, there were no (*P* > 0.05) treatment effects on d 35 the bursa and breast weight.

**Table 4:** Effect of single strain *B. subtilis* direct fed microbial (BSDFM) supplementation in drinking water on organ weights and jejunal histomorphology in broiler chickens fed a corn and soybean meal-based diet challenged with *Eimeria* (postchallenge period).

	Interaction effects					Main effects					P-values		
BSDFM^1^	-		+			BSDFM		*Eimeria*					
*Eimeria*^2^	-	*+*	-	*+*	SEM	-	*+*	-	+	SEM	BSDFM	*Eimeria*	BSDFM*Eimeria
Organ weight (g/kg BW)													
Liver													
d 14	29.44	31.74	28.92	31.79	0.55	30.59	30.36	29.18^b^	31.76^a^	0.39	0.672	<0.001	0.607
d 35	23.62	23.95	24.82	24.10	1.65	23.79	22.96	24.22	22.53	1.16	0.620	0.314	0.231
Spleen													
d 14	1.05	1.05	1.00	1.04	0.02	1.05	1.02	1.02	1.05	0.01	0.516	0.558	0.645
d 35	1.00	1.22	1.10	1.22	0.06	1.11	1.16	1.05^b^	1.22^a^	0.04	0.414	0.011	0.427
Bursa													
d 14	1.71^ab^	1.81^a^	1.56^b^	1.93^a^	0.06	1.76	1.74	1.63^b^	1.87^a^	0.02	0.773	<0.001	0.039
d 35	1.40	1.45	1.42	1.63	0.09	1.43	1.52	1.41	1.54	0.07	0.308	0.186	0.391
Breast													
d 35	220.8	211.0	220.6	214.9	7.36	215.9	217.7	220.7	213.0	4.34	0.765	0.220	0.743
Jejunal Histomorphology d 14													
Villi height (VH), µm	913.1	863.2	1031.5	879.3	57.04	888.1	955.4	972.3	871.2	40.35	0.251	0.090	0.380
Crypt depth (CD), µm	154.3	167.2	157.2	142.7	11.42	160.8	149.9	155.7	155.0	8.09	0.355	0.947	0.244
VH: CD	6.01	5.07	6.59	6.17	0.241	5.54^b^	6.38^a^	6.30^a^	5.62^b^	0.17	0.002	0.010	0.297

a,b,c Within a row, LSMeans assigned different letters differ, *P* < 0.05.

1 +BSDFM birds received 2 mL of broth containing 10^8^ CFU of BSDFM per mL in drinking water daily from d 0 to 13.

2 *Eimeria* birds received 1 mL of saline solution with 25,000 *E. maxima* and 100,000 *E. acervulina oocysts (+Eimeria)* or the same solution without *Eimeria (-Eimeria)* on d 9.

**Figure 2:**
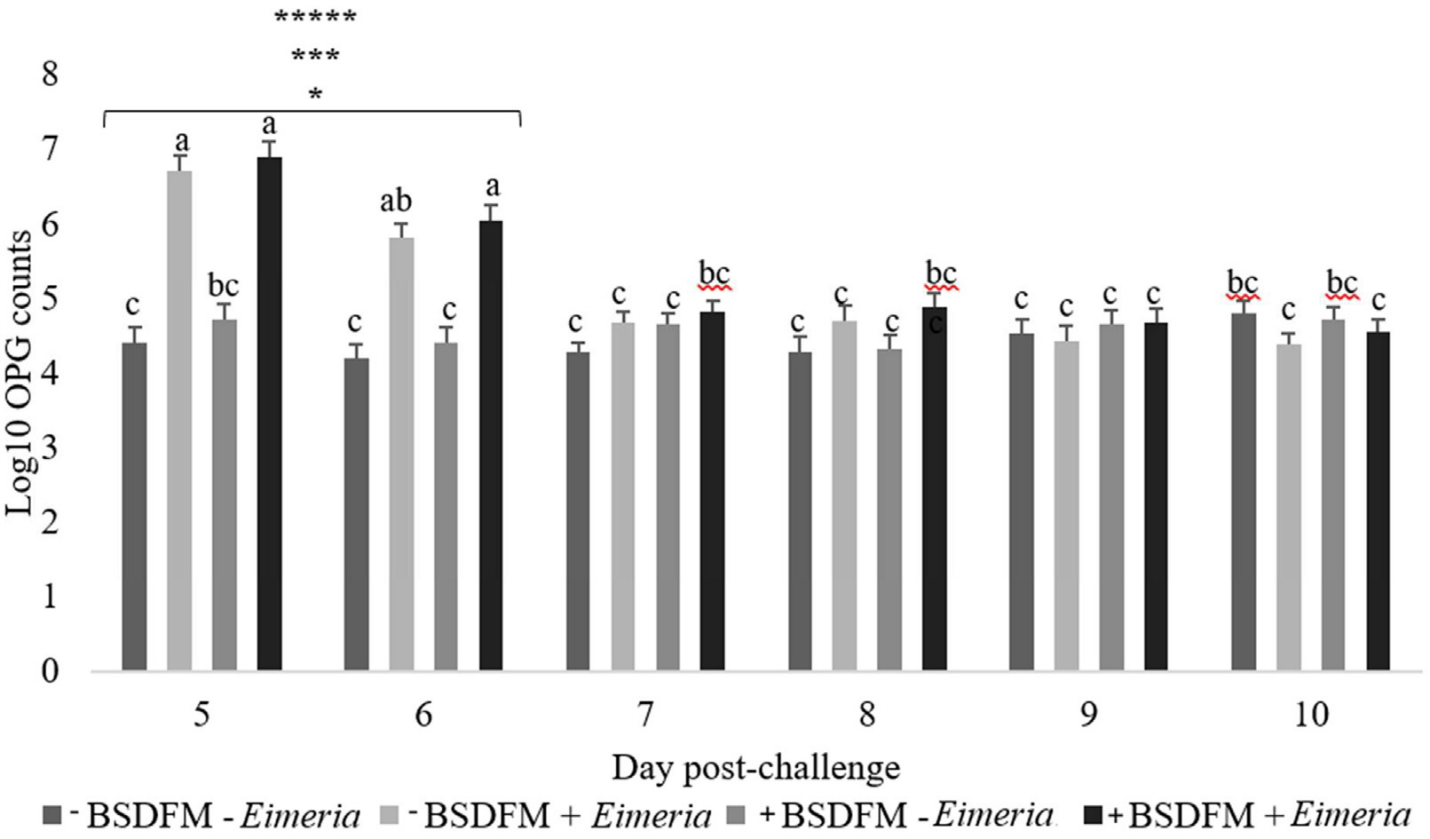
Effect of single strain *B. subtilis* direct fed microbial (BSDFM) supplementation in drinking water on oocysts shedding per gram (OPG) in broiler chickens challenged with *Eimeria.*^1^+BSDFM birds received 2 mL of broth containing 10^8^ CFU of BSDFM per mL in drinking water daily from d 0-13. ^2^*Eimeria* birds received 1 mL of saline solution with 25,000 *E. maxima* and 100,000 *E. acervulina oocysts (+Eimeria)* or the same solution without *Eimeria (-Eimeria)* on d 9. *P*-values were < 0.05 were designated as; ***** for day postchallenge x *Eimeria*; **** for BSDFM x *Eimeria*; *** for day postchallenge; ** for BSDFM; * for *Eimeria.*^a,b,c^Within a row, LSMeans assigned different letters differs, *P* < 0.05.

There were no (*P* > 0.05) interactions between BSDFM and *Eimeria* or the main effects of BSDFM and *Eimeria* challenge on VH, CD and VH: CD ratio (Table 4). However, there was a tendency (*P* = 0.099) for *Eimeria* to decrease VH. Regarding the VH: CD, the main effects were such that the *Eimeria* reduced (*P* = 0.01) the VH: CD by approximately ∼11.5% whereas BSDFM improved (*P* = 0.002) VH: CD by ∼14%. There were no (*P* > 0.05) interactions between BSDFM and *Eimeria* on duodenal and jejunal lesion scores at 5 d postchallenge (data not shown). Though biosecurity plans were in place, two birds in the unchallenged BSDFM group indicated intestinal lesions. However, *Eimeria* birds had higher (*P* < 0.05) duodenal and jejunal scores and there was no effect (*P* > 0.05) of BSDFM on lesion scores (data not shown). Regarding the oocyst count, there was a significant interaction between the *Eimeria* challenge and day (*P* < 0.001), such that at 5 and 6 DPI, challenged birds had higher (*P* < 0.001) OPG (Figure 2).

## DISCUSSION

Though *Bacillus* based probiotics differ in their efficacy, there are varying reports on their ability to improve broiler growth performance via numerous modes of action. These include pathogen inhibition, modulation of GIT microbial ecology, contributing to host metabolism, and enhancing immune responses (Lee et al., 2015; Neijat et al., 2019a,b,c; Bilal et al., 2021; Sandvang et al., 2021; Xu et al., 2021). Three crucial stages characterize the life cycle of *Eimeria* species (multiplication and sexual reproduction in the intestine and sporulation in the external environment) (Chapman, 2014; Chapman et al., 2016). The sporulated oocysts contain eight sporozoites, invade the enterocytes for parasitic feeding and multiple asexual divisions to form the schizont or meront full of merozoites. After 3 to 4 d post-challenge, the fully developed schizont bursts open and sets free the fusiform-shaped merozoites; these merozoites possess an apical complex, allowing them to move and invade the intestinal epithelial cells, leading to the formation of subsequent schizont generations through asexual reproduction (Mesa-Pineda et al., 2021). As such, the deleterious effects from an *Eimeria* challenge are seen in 3 to 4 d postchallenge.

As a physiological response to infection, FI and WI intake has been demonstrated to decrease from 4 to 5 d following *E. acervulina* and *E. maxima* challenge with FI exhibiting considerable reduction (Reid and Pitois, 1965). Reduced growth performance from a similar challenge dose of *E. maxima* and *E. acervulina* was also reported in our previous studies using the same model (Kim et al., 2017; Leung et al., 2019a,b; Lu et al., 2019). Intestinal inflammation triggered by *Eimeria* invasion damages the intestinal epithelium, impairing barrier, and absorptive functions, and as such increasing the host energy demands for immune functions, effectively diverting energy away from growth. Compensatory gain, in which *Eimeria*-challenged birds reach a similar final BW to nonchallenged birds can occur following an experimental *Eimeria* challenge (Gautier et al., 2020). An anomaly in our findings was an improvement in FCR in d 20 to 29 (10–20 post-challenge). This might indicate compensatory gain to recover from losses experienced during the challenge. However, if the intestinal damage of *Eimeria* replication is sufficient, along with the other pre-disposing conditions for *C. perfringens* proliferation in the absence of a gut modulator (i.e., AGP), NE can arise and reduce the final market BW of the flock (Abd El-Hack et al., 2022).

Although BSDFM did not improve FCR due to increased FI, BSDFM increased BW and BWG. *B. subtilis* B2A isolated from soil and selected due to its inhibition of *Salmonella typhimurium* had no effect on growth in broiler chickens but lowered FI and improved FCR in linear fashion up to 1.0 × 10^6^ CFU/g feed (Park and Kim, 2014). Increased growth was reported by Šimunović et al. (2022), in birds provided with *B. subtilis* PS-216 at 2.5 × 10^6^ CFU/mL in the drinking water for 8 or 21 d. Thus, two phylogenetically distinct *B. subtilis* strains can have differing abilities to improve growth performance across different genetic breeds and rearing conditions (Rhayat et al., 2017). Conversely, adverse reports of *B. subtilis* on broiler chicken growth performance have been reported. *B. subtilis* PB6 provided at 1.1 × 10^5^ CFU/g feed by Wang et al. (2018) to birds given an *Eimeria* vaccine at hatch had decreased FI on d 0 to 14 and 15 to 28 and BWG on d 15 to 28 and increased mortality on d 15 to 28 compared to the negative control. Differences in the ability of *B. subtilis* strains to improve broiler growth performance may be due to differences in the dose given, method of administration (i.e., formulated in feed *vs*. added to drinking water), length of administration, experimental design, the intrinsic probiotic properties of the strains (i.e., production of enzymes, AMP) and their ability to colonize the GIT (Park and Kim, 2014; Rhayat et al., 2017; Wang et al., 2018; Šimunović et al., 2022). There are differing reports in the literature regarding the potential of *B. subtilis* BSDFM products to mitigate the adverse effects of an *Eimeria* challenge. Ross 708 broilers raised on used litter and given *B. subtilis* (Avicorr) at 1.5×10^5^ CFU/g feed for 28 d reported no improvements in growth performance throughout the trial; however, *B. subtilis* had immunomodulatory effects, decreasing *Eimeria*-specific antibodies in the serum (Lee et al., 2014). A follow-up study by the same authors provided the same dose and strain of *B. subtilis* BSDFM; however, they selected it from agricultural sources based on its inhibitory ability against different avian pathogens (Lee et al., 2015). These authors reported that *B. subtilis* supplementation during unfavorable growth conditions (i.e., used litter) positively modulated the immune response, enhancing broiler growth at d 14 and 28, marked by increases in BW compared to the unchallenged control (Lee et al., 2015). Coccidiosis and later NE models have reported the beneficial effects of *B. subtilis* inclusion on broiler growth performance (Rhayat et al., 2017; Whelan et al., 2019; Wang et al., 2021). Wang et al. (2021) reported that *B. subtilis* (DSM29784) did not affect growth performance in starter or growth phases; however, BSDFM improved d 43 to 63 BW and d 1 to 63 average daily BWG in birds challenged with *Eimeria* on d 15 followed by *Clostridium perfringens* on d 18 to 21. *Clostridium perfringens* challenge caused a 16% decrease in BWG and increased FCR by 19% compared to the control, while the provision of *B. subtilis* improved both performance parameters to be similar to that of non-challenged control (Rhayat et al., 2017). The effects of the tested strain in a *C. perfringens* challenge model in poultry should be further explored basis in vitro tests revealed the strain created an inhibitory zone around *C. perfringens* when co-cultured (unpublished data). Moreover, the BSDFM strain used in the current study is a superior producer of cellulase and xylanase compared to the control (ATCC 6,633) and successfully fermented soybean meal to improve the nutrient digestibility for swine (Akhtar et al., 2022). Future studies should investigate the effect of dose on FI and FCR, as well as its impact on the digestive enzyme activity of broiler chickens as a mechanism of action behind improved growth performance.

Increased inflammation and immune organ hypertrophy following an *Eimeria* challenge, combined with decreased growth, have been reported. Poudel et al. (2021) stated that *Eimeria* challenge on d 14 increased d 27 spleen weight; however, the effects were gone by d 36. Additionally, the *Eimeria* challenge decreased the absolute bursal weight; however, the challenge also reduced BW, and the relative bursa weight was unaffected (Poudel et al., 2021). These authors also reported no effects of *B. subtilis* PB6 supplementation in feed on d 35 relative organ weights. Wang et al. (2018) gave *B. subtilis* for 54 d in feed and provided an *Eimeria* challenge on d 21. Neither treatment affected d 26 spleen nor bursal weights, but the challenge increased d 54 relative bursal weights, and a later study reported no effects from *B. subtilis* or d 14 *Eimeria* challenge on spleen weights (Wang et al., 2019b). The Bursa of Fabricius is an immune organ responsible for T and B cell maturation and can be used to assess the immune status of a bird (Park and Kim, 2014). Mohamed et al. (2022) reported that *B. subtilis* (ATCC 19659) at 3 × 10^8^ CFU/g feed increased d 21 bursa weight, furthermore, Park and Kim (2014) noted increased bursa weight in response to a *B. subtilis* (B2A) dose of up to 1 × 10^6^ CFU/g. These varying results may be due to the interactive effects between the specific *B. subtilis* strains and *Eimeria* challenge, the dose, the method of application, and the experimental design.

The endogenous phase of *Eimeria* oocysts causes structural changes in the morphology and reduces the intestinal absorptive area (Mesa-Pineda et al., 2021). Marked decreases in VH and the VH: CD have been reported following oral *Eimeria* inoculation in broilers (Kim et al., 2017; Leung et al., 2019a, Leung et al., 2019b; Poudel et al., 2021). Increased intestinal surface area (i.e., the VH: CD) within the GIT facilitates greater nutrient absorption and translates to improved growth performance in broiler chickens (Jayaraman et al., 2017; Rivera-Perez et al., 2021). Poudel et al. (2021) reported no effects of *B. subtilis* on histomorphology; however, they stated that an *Eimeria* challenge increased CD and a reduced VH: CD in both the ileum and jejunum. Improvements in growth performance in the current study from *B. subtilis* supplementation may result from the increased intestinal surface area; conversely, the lack of gains in growth performance in the study by Poudel et al. (2021) may be explained by the lack of effects of supplemented *B. subtilis* on intestinal morphology. Previous work in swine epithelial cells suggested that the BSDFM strain tested in the current study can protect against ETEC-induced inflammation, improved barrier function, and cell proliferation rates, and reduced apoptosis (Sudan et al., 2022). However, the specific metabolite production, effect on tight-junction protein gene expression, and safety (i.e., cytotoxicity) of the strain still need to be tested in avian tissues and with avian-specific enteric pathogens, such as *Eimeria spp*. and *C. perfringens*.

Lesion scores quantify the visible damage to validate that the challenge model successfully induced coccidiosis. Reports of *E. maxima* and *E. acervulina*- induced lesions in the duodenum and jejunum are consistent with similar challenge models in our laboratory. For example, Kim et al. (2017) (d 5; 1mL 25,000 oocysts *E. acervulina*, 6,000 oocysts *E. maxima*) and Leung et al., 2019a, Leung et al., 2019b (d 10) 1) high-dose 100,000 oocysts *E. acervulina*, 60,000 oocysts *E. maxima* oocysts; 2) low-dose 25,000 oocysts *E. acervulina*, 5,000 *E. maxima*). *Eimeria* lesions cause nutrient malabsorption and induce an inflammatory state, decreasing host immune status and barrier function and increasing susceptibility to intestinal dysbiosis. Increased oocysts shed on d 5 and 6 post-challenge in the current study align with increased shedding of *E. maxima* and *E. acervulina*. Future studies should explore the impact of different doses of BSDFM based on the tested strain on *Eimeria* replication and later NE. Additionally, challenge birds should be housed in a separate experimental room to minimize potential cross-contamination.

*Bacillus subtilis* based DFM are promising interventions for the animal production industry due to their ability to withstand harsh environmental stressors to produce numerous beneficial compounds within the GIT. Enteric pathogen control by *Bacillus subtilis* based DFM products may offer an alternative to AGP for reducing NE onsets and ensuring broiler chickens reach their intended growth potential. The current study was the first to test single strain BSDFM (BS-9) in poultry; future studies should explore the effects of varying doses on broiler chicken endogenous enzyme production, immune response, the microbiome, and its incorporation into animal feed and ensure additional biosecurity measures are in place.
